# Strategic valorization of invasive alien plants: A bioeconomic review for sustainable product development

**DOI:** 10.3389/fpls.2025.1697102

**Published:** 2026-01-13

**Authors:** Pragati Patil, M. P. Divya, K. T. Parthiban, A. Balasubramanian, S. Varadha Raj, R. Ravi, R. Ashick Rajah

**Affiliations:** 1 Forest College and Research Institute, Tamil Nadu Agricultural University, Mettupalayam, India; 2 Directorate of Research, Tamil Nadu Agricultural University, Coimbatore, India

**Keywords:** bioeconomy, invasive alien plant species, prioritization, resource valorization, value addition

## Abstract

Invasive alien plant species (IAPS) pose serious ecological and economic threats due to their aggressive proliferation and disruption of native ecosystems. However, their high biomass yield and rich phytochemical profiles offer significant potential for value-added utilization within circular bioeconomy frameworks. This review evaluates five major IAPS viz., *Lantana camara*, *Prosopis juliflora*, *Leucaena leucocephala*, *Acacia mearnsii*, and *Senna* sp*ectabilis* for their suitability in bioenergy, pulp and paper, natural dye production, pharmaceuticals, compost, and engineered wood. Quantitative assessments using multi-criteria scoring, pharmacological activity heatmap, and biomass-to-product flow models reveal that *P. juliflora* is the most versatile species, showing high performance across all categories, while *L. camara* and *L. leucocephala* emerge as specialized candidates for dyes, pharmaceuticals, and fodder applications, respectively. *S.* sp*ectabilis* exhibits biochar and soil improvement potential, and *A. mearnsii* demonstrates value in pulp and water purification. Despite technical and regulatory challenges, the strategic valorization of IAPS can simultaneously advance ecological restoration and green economic development. The article emphasizes integrative approaches and policy support for mainstreaming IAPS-based resource management.

## Introduction

Invasive alien plant species (IAPS) are non-native plants introduced either intentionally or unintentionally beyond their natural geographic range. Once established, these species exhibit rapid growth and aggressive reproduction, often outcompeting native vegetation. Their proliferation is supported by the absence of natural predators and pathogens in the introduced environment, which results in significant ecological, economic, and social consequences (Malongweni et al., 2025). Notable examples such as *Lantana camara*, *Parthenium hysterophorus* (Congress grass), and *Eichhornia crassipes* (water hyacinth) have severely disrupted ecosystems, reduced biodiversity, and impaired agricultural productivity and livelihoods. They also alter species interactions, deplete water resources, and increase the risk of natural disasters like wildfires (Brewington et al., 2024). The economic burden of managing IAPS amounts to billions of dollars annually due to their adverse effects on agriculture, infrastructure, and natural ecosystems (Eschen et al., 2021).

**Figure 1:**
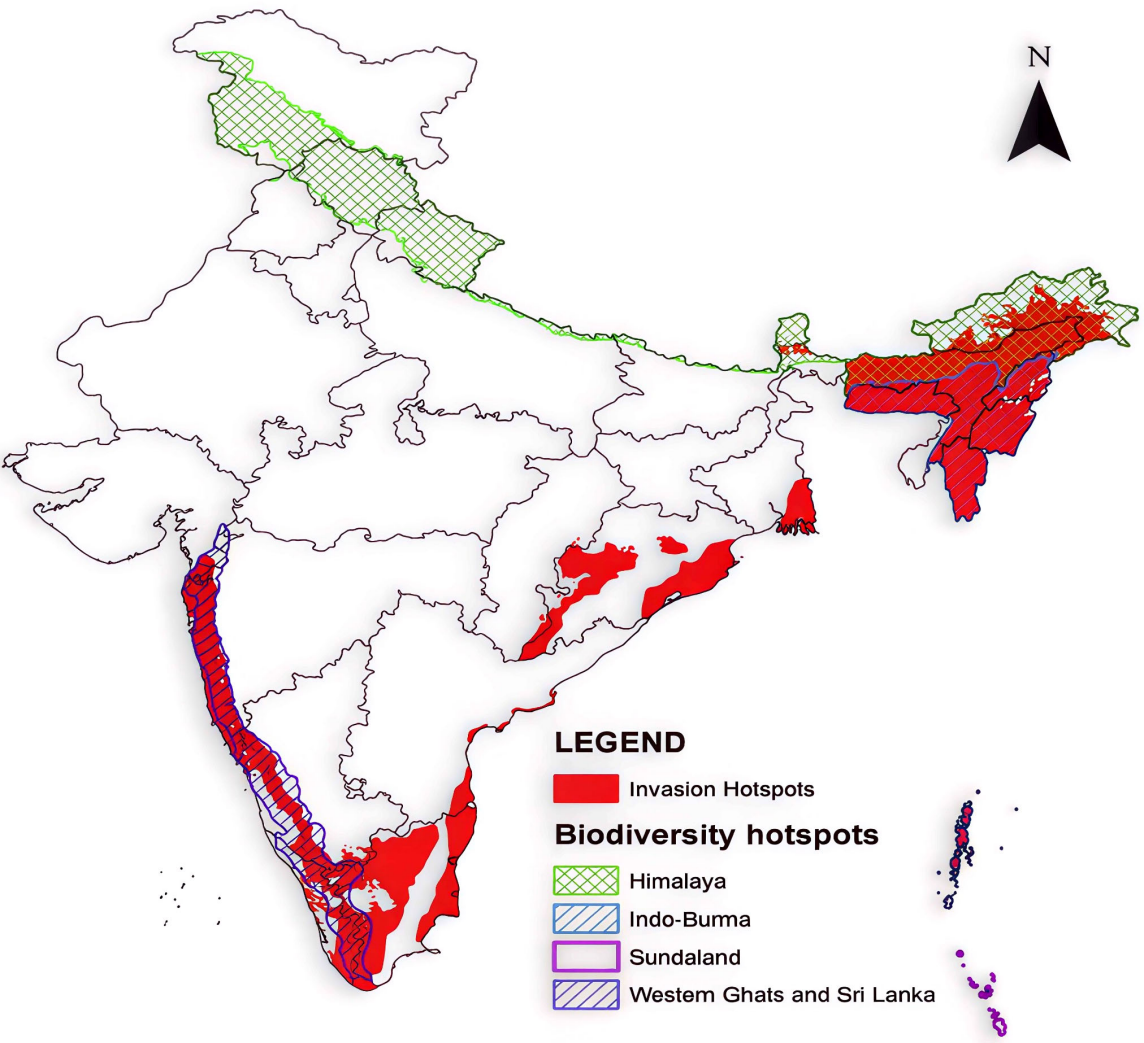
Geographic distribution of major invasive plant species invasion hotspots across India. This map visually represents areas significantly impacted by invasive alien plant species, highlighting regions where their proliferation poses substantial ecological and economic challenges. Data adapted from TN PIPER (2022).

Invasive alien plant species (IAPS) have extensively impacted diverse ecosystems globally and in India, causing significant ecological and economic challenges (Rai and Singh, 2021; Kahrić et al., 2022). Globally, IAPS like *L. camara*, *Eichhornia crassipes* (water hyacinth), and *A. mearnsii* have invaded millions of hectares of forests, wetlands, grasslands, and agricultural lands, with an estimated global economic cost exceeding $70 billion annually. In South Africa, for instance, *A. mearnsii* has invaded over 2.5 million hectares (Lusizi et al., 2024), while *Eichhornia crassipes* covers over 10,000 hectares of Lake Victoria, disrupting water systems and livelihoods. In India, over 1.5 million hectares of forests are affected by species like *L. camara* and *Chromolaena odorata*, with *Lantana* alone invading 44% of tiger reserves (Rastogi, 2022). Wetlands, such as Kerala’s Vembanad Lake, see *Eichhornia crassipes* covering 20% of its surface, while *P. juliflora* dominates 15 million hectares of arid lands, notably in Rajasthan and Gujarat. Bang et al. (2022) reported that the Indian economy would suffer US$ 116 billion per year because of IAS. The spread of IAPS in India leads to crop yield losses up to 40%, health hazards, and an annual economic burden of ₹6,000 crores on management, underscoring the urgent need for mitigation and sustainable utilization strategies. In India, the proliferation of species such as *Lantana camara*, *Prosopis juliflora*, and *Chromolaena odorata* has significantly affected forest ecosystems, agricultural land, and water bodies (Munda et al., 2024). As shown in (**Table 1**), *Lantana camara* and *Prosopis juliflora* represent the most widespread invasive plant species in Tamil Nadu, accounting for a substantial portion of the total invaded area (TN PIPER, 2022). To illustrate the national scope of the invasive alien plant species challenge, (**Figure 1**) provides a geographic overview of major invasion hotspots throughout India (TN PIPER, 2022).

**Table 1:** Estimated Area and Percentage of Major Invasive Plant Species in Tamil Nadu.

Species	Area (ha)	Percentage (%)
*Acacia mearnsii*	22400	7.04
*Chromolaena odorata*	11532	3.63
*Eucalyptus sp.*	6780	2.13
*Lantana camara*	185000	58.18
*Parthenium hysterophorus*	12150	3.82
*Prosopis juliflora*	56000	17.61
*Senna spectabilis*	2400	0.75
*Opuntia sp.*	2300	0.72
*Pinus sp.*	2700	0.85
Others	16738	5.26
Total	318000	100

Data adapted from TN PIPER (2022), based on Tamil Nadu Forest Department (TNFD).

Utilizing IAPS for value-added products offers a sustainable solution that addresses both ecological challenges and economic opportunities. The transformation of IAPS into value added products like biofuels, natural dyes, pharmaceuticals, paper, and bioplastics, can enable the conversion of these ecological threats into valuable resources. This approach aligns with global goals of resource efficiency, circular bio-economy, and sustainable development. The objectives of this article are to analyze the potential of IAPS (*L. camara*, *P. juliflora*, *L. leucocephala*, *A. mearnsii* and *S.* sp*ectabilis*) for value-added applications, highlight innovative solutions for their utilization, examine challenges in this domain, and propose strategies to integrate their management into sustainable resource frameworks.

## Bioenergy applications

Studies have demonstrated that *L. camara* biomass, when bioaugmented with cellulolytic bacteria and cattle dung, produced 950–980 L/kg of volatile solids of biogas with 57–60% methane, while NaOH pretreatment further increased biogas yield to 13.4 L/100 g (Sinha et al., 2021). Autoclave pretreatment on *L. camara* significantly enhanced solubilization and methane production to 3656 mL in 5 weeks (Saha et al., 2023). A study on *L. camara* stem revealed that 1% (v/v) H_2_SO_4_-assisted autoclaving was the most effective pre-treatment, yielding a total of 196.0 mg/g of fermentable sugars from raw biomass (Kumar et al., 2020). Comparative assessments confirmed *L. camara* (12%) as a more effective ethanol feedstock than *I. balsamina* (10%) and *R. communis* (7%) (Wijekoon et al., 2024). Briquettes made from *L. camara* and *P. juliflora* reached a density of 1.2 g/cm^3^ and energy density of 23.05 GJ/m^3^ (Kumar and Chandrashekar, 2020). The briquettes made using *L. camara* with optimal blends of *Bambusa bambos* and sawdust improved their physical properties (Patil et al., 2023). *L. camara* is a suitable blender with other raw materials in briquetting technology. *P. juliflora* wood charcoal showed high calorific value of 32.8 MJ/kg at 600 °C and a yield of 1–2 kg per 10 kg of green wood (Kumar and Chandrashekar, 2020). Its favorable composition which includes 25% fixed carbon, low ash, and high lignin (25%) (Ram, 2020) makes it ideal for traditional and commercial charcoal production. Walter and Armstrong (2014) estimated that a medium-sized charcoal production unit can process around 240 tons of *P. juliflora* per month, with the production of 40–50 tons of charcoal with a market price of Rs.5000 per ton. *A. mearnsii* biomass also demonstrated strong calorific potential, ranging from 4572 to 5408 kcal/kg depending on tree part and age (da Silva et al., 2017), with modern metal kilns yielding 120–150 kg of high-quality charcoal per batch (Charcoal team, 2023). In Rio Grande do Sul State, Brazil, *A. mearnsii* is grown in rotational cycles of 5 to 6 years, with its harvested wood primarily used for charcoal production and fuelwood (Tiruneh et al., 2024). Ayaa et al. (2022) found that *S.* sp*ectabilis* exhibited the highest cellulose content (48%) and heating value (17.84 MJ/kg) among six IAPS, indicating suitability for pyrolysis-based biochar and bio-oil production. *L. leucocephala* is another notable fuelwood species, offering 4434 kcal/kg energy, low ash (1.15%), and high volatile content (79.6%) (Chavan et al., 2015). Its seeds, containing 5–8% oil, can be used as a feedstock for biodiesel production (Yusuff et al., 2019), and the residual cake can be utilized as biofertilizer or biocide. These findings underscore the bioenergy potential of IAPS and their potential to replace conventional biomass resources in sustainable energy systems.

## Pulp and paper potential

Invasive alien plant species (IAPS) offer an alternative and sustainable source of lignocellulosic biomass for the pulp and paper industry, especially given the increasing pressure on conventional forest-based raw materials. *L. camara* exhibits holocellulose content between 66.6% and 70.5% with moderate lignin (27%) and pentosan levels, making it compatible with alkaline pulping (Bhodiwala et al., 2023). *P. juliflora* shows excellent pulping performance with up to 96.9% holocellulose, 43.3% α-cellulose, and low ash content (2.12%), resulting in strong pulp yield and good fiber length (1.20 mm) (Elkhalifa, 2020). The pulping suitability of *L. leucocephala* is well supported by its high holocellulose content, which typically ranges between 68.5% and 74.5%. A work done by (Pandey et al., 2022) has shown that combining chip de-structuring with xylanase pretreatment improves kraft cooking performance, yielding 48.2–50.1% pulp while lowering the kappa number to 18.6–23.7. Its fiber characteristics such as mean fiber length of 1.15 mm, fiber diameter of 23.66 µm, and wall thickness of 3.63 µm, together with a Runkel ratio of 0.44 and flexibility ratio of 0.69 indicate fibers that collapse easily and bond effectively, traits closely associated with strong tensile and tear behavior in paper sheets (Khan et al., 2023). These observations align with recent chemical composition studies reporting holocellulose values near 70%, further confirming the species’ promise as a reliable and efficient pulpwood resource (Sivan et al., 2023). Kraft pulping of *A. mearnsii* bark and wood has yielded (52.6–53.5%) high-strength paper with degrees of polymerization exceeding 2800- 3690 (Neiva et al., 2024). At the industrial level, companies like Tamil Nadu Newsprint and Papers Limited (TNPL) have incorporated *S.* sp*ectabilis* in blended pulps, successfully launching blue-tinted paper under the ‘OPAL’ brand (Thepulpandpapertimes.com, 2024). The pulping trials across these species show acceptable kappa numbers, high cellulose recovery, and suitable physical properties for packaging, writing, and printing applications. Their rapid growth on marginal lands and year-round availability reduce raw material shortages and support low-input cultivation models. Overall, IAPS-based pulp production represents a viable, eco-friendly alternative that contributes to both sustainable paper manufacturing and invasive species control.

## Natural dyes

Studies have increasingly recognized invasive alien plant species (IAPS) as promising sources of natural dyes with applications in textiles, cosmetics, eco-friendly inks and sustainable industrial applications. *L. camara* flowers and leaves produce vibrant pigments ranging from yellow to dark orange, which have shown good fastness properties and antimicrobial activity when applied to cotton and silk fabrics (Datta et al., 2023). Dhanush et al. (2020) optimized dye extraction using a Box-Behnken Design. Optimal conditions included 92°C for 174 minutes (MLR 2:100) for flowers and 79°C for 36 minutes (MLR 3:100) for leaves, yielding 29.7% and 26.2% dye respectively. The extracted dyes contain flavonoids, carotenoids, and anthocyanins that contribute to their coloration and functional bioactivity. *P. juliflora* heartwood and bark have been utilized to produce reddish-brown and orange hues (Odero et al., 2021), with successful applications not only in traditional textiles but also as sensitizers in dye-sensitized solar cells (DSSC), where they demonstrated the highest photo-conversion efficiency (0.322%) (Sowmya et al., 2021) and antibacterial coating properties when applied on a polyester fabric (Kumar et al., 2022). Recent phytochemical work shows that *S.* sp*ectabilis* contains anthraquinone pigments. These compounds are responsible to produce yellow–orange hues in many natural dyes that highlights its clear dye potential, although their industrial exploitation remains limited compared to *L. camara* and *P. juliflora* (Selegato et al., 2017; Alegbe and Uthman, 2024). Various parts of *L. leucocephala* including pods, leaves, and bark are known to yield natural dyes in red, brown, and black tones, due to high tannin content (Bakewell-Stone, 2023). Priyanti et al. (2021) demonstrated *L. leucocephala*’s peel extracts to dye cotton effectively even with low mordant concentrations (0.5–0.6%). *A. mearnsii* bark, widely known for its high tannin content and serving as a natural mordant in dyeing processes, improves dye fixation and enhancing color vibrancy (Ogawa and Yazaki, 2018). The valorization of IAPS-derived dyes addresses the growing demand for non-toxic, biodegradable alternatives to synthetic dyes, offering significant ecological and commercial benefits while contributing to the integrated management of invasive plant populations.

## Medicinal and pharmaceutical uses

The bioactive potential of invasive alien plant species (IAPS) has gained increasing attention for pharmaceutical and therapeutic applications, owing to their rich secondary metabolite profiles. Extracts from *L. camara* exhibit significant antimicrobial, anti-inflammatory, and wound-healing activities (Singh, 2023), attributed to compounds such as oleanolic acid, lantadenes, and flavonoids. Traditionally, *L. camara* is used as a sudorific, intestinal antiseptic, diaphoretic, and treatment for tetanus, rheumatism, and malaria (Firdaus et al., 2024). Pounded leaves are applied to cuts, ulcers, and swellings, while decoctions of leaves and fruits are used externally for skin conditions and internally for fevers (Khandagale et al., 2024). Seeds contain 9% oil, rich in fatty acids like linolenic, linoleic, oleic, stearic, and palmitic acids. Roots are a source of oleanolic acid, which has anti-inflammatory, antioxidant, hepatoprotective, and anti-tumor properties (Woldeamanel et al., 2022). Recent investigations highlight growing pharmacological interest in *A. mearnsii*. A 12-week randomized trial involving 68 participants showed that daily intake of its bark-derived proanthocyanidins produced a significant reduction in visceral fat area compared to placebo (Baba et al., 2024). Analytical profiling indicates that roughly 77% of the bark extract consists of oligomeric proanthocyanidins with a degree of polymerization between 1 and 11, supported by strong *in-vitro* inhibition of α-amylase and α-glucosidase (Chen et al., 2018). Tannin fractions also exhibit UV–vis absorption maxima at 283–287 nm and form composites with spent bark ash (around 4.5%) that retain stable functional characteristics (Bueno et al., 2025). Xiong et al. (2016) found phenols and flavonoids in *A. mearnsii* contribute to its antioxidant and anti-inflammatory effects. Ogawa and Yazaki (2018) noted the plant’s antitumor activities which suggests its possible applications in cancer therapy. *P. juliflora* holds great therapeutic potential. Traditional medicine utilizes its bark, leaves, pods, and seeds to treat a variety of ailments including stomach disorders, skin infections, wounds, and *eye* diseases (Saleh et al., 2023). Walter and Armstrong (2014) noted its use in expectorant syrups and lactation-boosting preparations. Its leaf and bark extracts display strong antibacterial properties against oral pathogens (Tarpale and Khamkar, 2025) and plant pathogens like *Xanthomonas campestris*, due to the presence of alkaloids and tannins (Jakatimath et al., 2021).

Although *S.* sp*ectabilis* is less explored, it has demonstrated notable anticonvulsant and antimicrobial activities. It is employed to treat rheumatism, insomnia, anxiety, and epilepsy, particularly in African and Asian regions (Selegato et al., 2017). Nuengchamnong and Suphrom (2023) further showed that flower extracts of *Cassia* sp*ectabilis* inhibit cholinesterase, supporting its neuropharmacological potential. Nkantchoua et al. (2018) demonstrated in mice that decoction of *S.* sp*ectabilis* protected against chemically and electrically induced seizures, delaying onset, reducing mortality, and even giving 100% protection against PTZ-induced convulsions. It is also used in Cameroon to treat epilepsy, constipation, insomnia, and anxiety.

**Figure 2:**
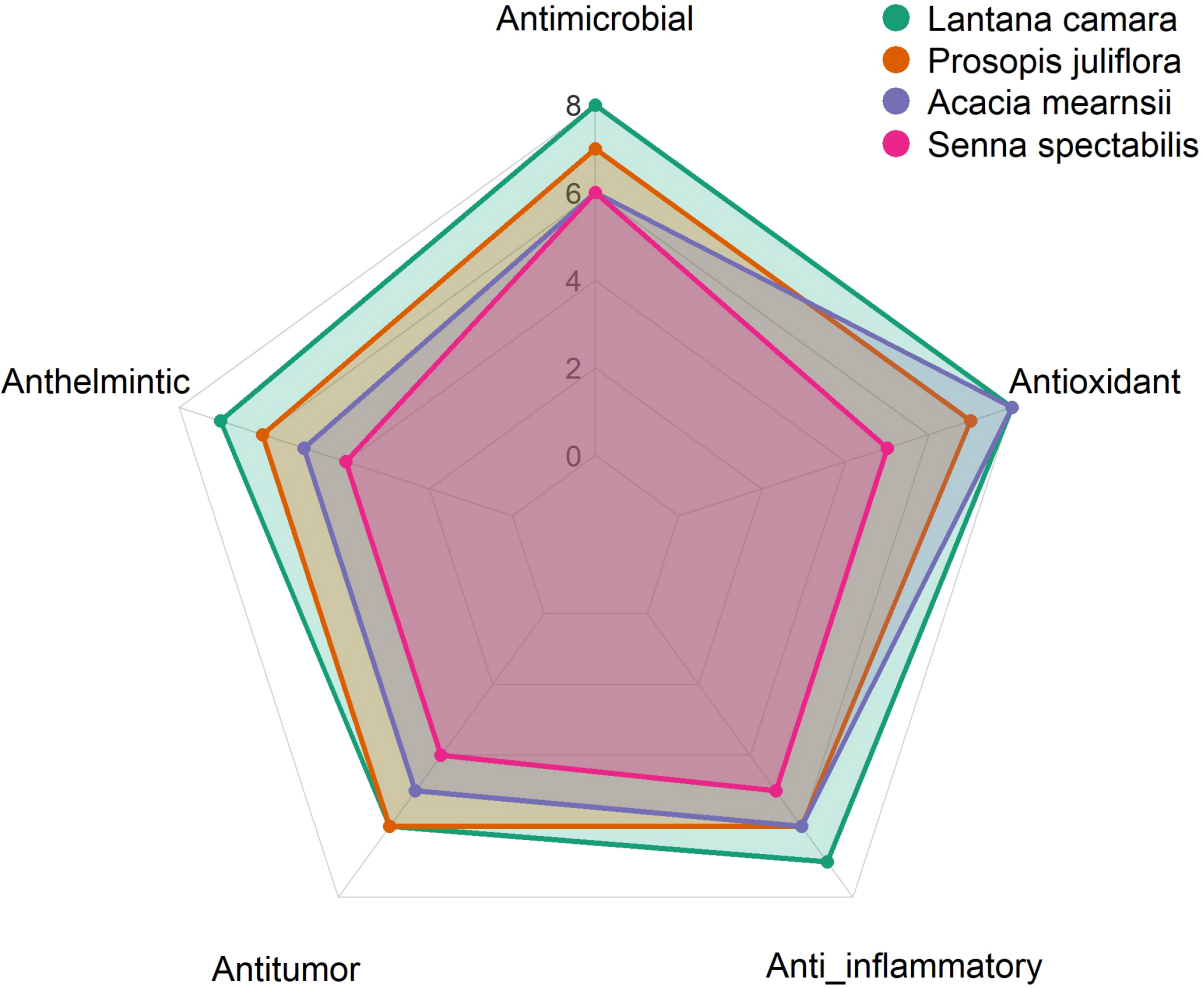
Comparative pharmacological activity of bioactive compounds derived from major invasive alien plant species. This radar chart provides a comparative analysis of five key pharmacological activities (Antimicrobial, Anthelmintic, Antitumor, Anti-inflammatory, and Antioxidant) for bioactive compounds derived from four major invasive alien plant species: *L. camara*, *P. juliflora*, *A. mearnsii*, and *S.* sp*ectabilis*. The chart visually represents the relative strengths of each species across these medicinal properties. *L. camara* shows high antimicrobial and antioxidant activity, *P. juliflora* exhibits strong antimicrobial, anti-inflammatory, and antitumor effects whereas *A. mearnsii* demonstrates high antioxidant potential. *Leucaena leucocephala* is excluded from this comparative radar due to insufficient pharmacological data available in the reviewed literature.

The seeds of *L. leucocephala* are utilized for their gum, which shows potential in anti-diabetic formulations and tablet-binding agents. Khalid et al. (2024) reported broad-spectrum antimicrobial, antidiabetic, and anthelmintic effects. The seed oil has also been used in pharmaceutical engineering for drug permeability modeling. The plant is reported to be an anthelmintic (Sriasih et al., 2025). Despite its toxic mimosine content, *Leucaena* is consumed in parts of Central America, Indonesia, and in Thailand either raw or processed (Bakewell-Stone, 2023). Despite promising preliminary data, comprehensive pharmacological validation, toxicity studies, and standardization protocols remain necessary to facilitate the integration of IAPS-derived products into mainstream healthcare and pharmaceutical industries. A comparative analysis of the pharmacological activities of bioactive compounds derived from major invasive alien plant species is summarized in the radar chart presented in (**Figure 2**).

## Compost, biochar and activated carbon

The transformation of invasive alien plant species (IAPS) into compost, biochar, and activated carbon presents an effective strategy for sustainable biomass valorization and soil restoration. Biochar production through pyrolysis of IAPS has yielded materials with high surface area, porous structure, and stable carbon content, which is useful for carbon sequestration, soil amendment, and pollutant adsorption. Notably, pyrolysis of *S.* sp*ectabilis* at 650°C resulted in biochar with favorable physical and chemical properties, supporting its use in agricultural and environmental applications. Diaz-Uribe et al. (2022) studied biochar from *P. juliflora* seed waste at 300°C, 500°C, and 700°C. Biochar yield was highest (34.12%) at 300°C, while the best dye removal efficiency (69%) occurred with material produced at 500°C. The porous structure (6–28 µm) facilitated chemisorption of methylene blue. Ali et al. (2023) applied *P. juliflora* biochar in maize and wheat fields that showed improved seed germination, shoot/root growth, and yield performance. Biochar enhanced the physicochemical and biological properties of leaching-prone soils, making it a viable amendment for sustainable agriculture. Dzokom (2024) further reported that *P. juliflora* biochar alone produced crop yields equivalent to those from conventional NPS fertilizers. The application of *Lantana camara* biochar along with inorganic fertilizers has been shown to improve fodder yield and quality in oats. According to Choudhary et al. (2023), this treatment enhances plant height, tillering, nutrient availability, and water-holding capacity, and improves overall soil health.

Vasiraja et al. (2024) evaluated activated carbon derived from *P. juliflora* stems for methylene blue dye removal from wastewater. The material had a surface area of 158.1 m^2^/g and showed a maximum dye removal efficiency of 90.65% at a 120 mg dosage within 50 minutes which highlights its strong adsorptive capacity. Wan Ibrahim et al. (2021) evaluated activated carbon made from *L. leucocephala* wood for cadmium adsorption. Higher activation temperatures (up to 800°C) and NaOH treatment significantly increased surface area (up to 776 m^2^/g) and adsorption capacity. Yusuff (2019) used *L. leucocephala* seed pods to develop activated carbon for removing hexavalent chromium from aqueous solutions. The optimal removal occurred at 45°C, pH 6.0, and a contact time of 100 minutes, with a maximum adsorption capacity of 26.94 mg/g. Schultz et al. (2024) successfully converted *A. mearnsii* bark residues byproducts of the tannin industry into activated carbon using ZnCl_2_ chemical activation. This carbon effectively removed organic pollutants from water. Activated carbon produced from *P. juliflora* seed waste and *A. mearnsii* bark exhibits strong adsorption capacities for dyes and heavy metals, indicating potential use in wastewater treatment. The valorization of IAPS into compost and carbon materials not only mitigates biomass waste accumulation but also supports circular bioeconomy initiatives aimed at improving soil health and environmental remediation.

## Plywood and particle board

**Figure 3:**
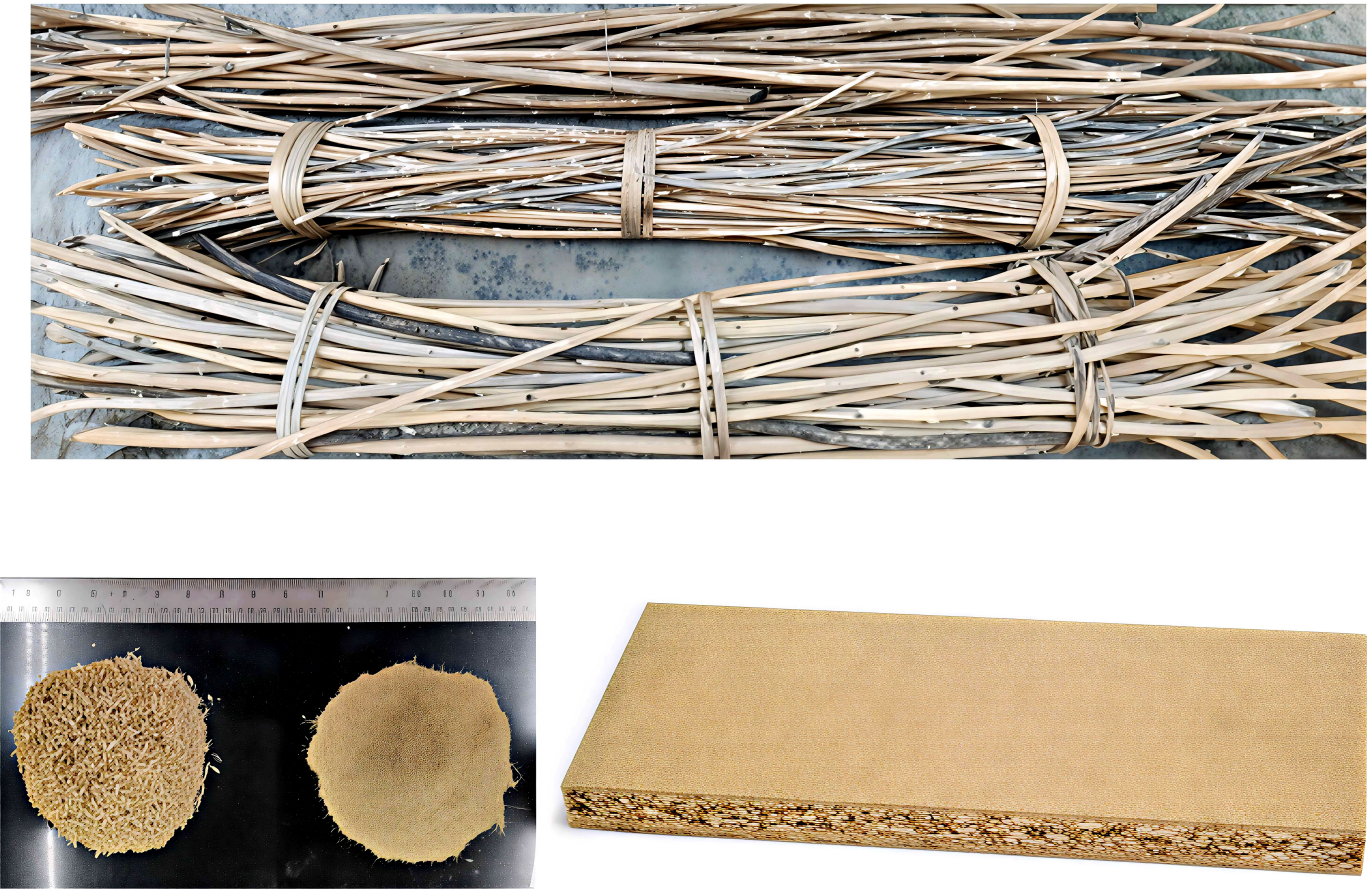
Example of a particle board manufactured using biomass from *Lantana camara*. This image demonstrates a tangible value-added product derived from *L. camara*, showcasing its potential for engineered wood applications. The production of such particleboards, meeting industrial specifications, has been successfully demonstrated (Ramkumar et al., 2023).

Studies have demonstrated that the dense lignocellulosic structure of certain invasive alien plant species (IAPS) offers considerable potential for engineered wood products such as plywood, particleboard, and fiberboard. The use of *Lantana* stalks of suitable sizes for furniture has been well studied, and it was reported that they may be used similarly to cane in furniture making (Ramkumar et al., 2023). As noted by Monika and Dhingra (2023), the termite-resistant and durable nature of *L. camara* has made it a viable raw material for the furniture sector. Soligas, South Indian tribal artisans, use *Lantana* as a substitute for rattan and turn it into value-added products such as furniture, toys, and other household utility items (Chatterjee, 2015). *Lantana* stalks are thin, crooked, tough, and long-lasting; thus they can be an excellent source of raw material for bio-composites. Ramkumar et al. (2023) demonstrated the successful production of three-layered particleboards using *L. camara* particles bonded with 8% urea-formaldehyde resin. These panels met the IS 3087 specifications for Grade-2 particleboard showing in (**Figure 3**). *S.* sp*ectabilis* is an excellent timber to make lightweight furniture and other wooden products. Its heartwood is brown, and the sapwood is whitish; the wood is heavy, soft, and hard, and has a good termite resistance. Although specific studies on its use in plywood or particleboard are limited, the wood’s density (450–750 kg/m^3^), machinability, and durability indicate potential suitability for engineered wood products. In several South American countries where *Prosopis* is indigenous, the wood is used extensively for furniture and floorings (Ruiz et al., 2022). Its use in composite materials has also been explored. Raj et al. (2020) developed bio-composites by incorporating *P. juliflora* wood flour into polylactic acid (PLA) matrices. An 80:20 PLA-to-fiber ratio yielded the best mechanical properties including enhanced tensile, flexural, and impact strength. Fine particle sizes improved reinforcement compared to coarse ones. Bhadewad et al. (2018) experimented with various resin concentrations in particleboard production using of *P. juliflora* wood. Their findings showed that varying resin content influences the mechanical and physical characteristics of the boards. In a study conducted by Rahman et al. (2020) on *L. leucocephala* was found to be suitable alternative for particle board manufacturing. All the boards passed EN312:2003 requirements suitable for interior use such as furniture in dry conditions.

## Herbicidal and insecticidal potential

*L. camara* leaf extracts possess potent insecticidal properties and could serve as natural alternatives to synthetic pesticides (Rajashekar et al., 2014). The allelopathic effect of *L. camara* L. has some beneficial impacts on Rice, as it increased chlorophyll content and grain yield while at the same time significantly reducing the growth of other associated weeds of rice; i.e. *Panicum psilodium* Trin. and *Commelina benghalensis* L (Bansal, 1998). In an experiment with rodents (*Mastomys coucha*) the crude extract from stem of *Lantana* by oral route shows antifilarial efficacy by killing 40% of adult *Brugia malayi* parasites and sterilizing three-fourths of the female worms (Al-Snafi, 2024). Extracts of *L. camara* can be used for protection of members of Brassicaceae family (e.g., mustard) against the aphid *Lipaphis erysimi* (Upadhyay et al., 2023). Extract of *L. camara* leaves (with 5% chloroform) was found significantly effective against termite worker (Patel and Narasimhacharya, 2024). An essential oil from its leaves possess adulticidal activity against the different mosquito species. Thus, this species can be utilized for oil-based insecticides as supplement to synthetic insecticides (Negi et al., 2019). Similarly, *L. leucocephala* releases mimosine, a toxic amino acid that inhibits the germination and early growth of competing plants even at low concentrations. Sahid et al. (2017) showed that mimosine increasingly inhibited weed growth (50%) against *Ageratum conyzoides*, *Emilia sonchifolia*, and *Tridax procumbens* weeds. Bio oil made from of *L. leucocephala* shows high insecticidal activity against the insect *Sitophilus oryzae* (L.) with a 30% mortality rate and approximately 97% after 7 days (Aly et al., 2023). Quy et al. (2024) reported that *L. leucocephala* plant extract suppressed the *Raphanus sativus* L. seed germination and plant growth with inhibition levels, 70% for seed germination, 91.46% for root length, 69.89% for stem length, 78.73% for fresh weight, and 49.53% for dry weight. Therefore, *L. leucocephala* extract could be a promising natural alternative for weed management. *P. juliflora* is a potential safe and environmentally friendly alternative for mite management. Under nursery conditions, *P. juliflora* aqueous leaf extract demonstrated effective control of *Tetranychus bastosi* on *Jatropha curcas*, with LC_50_ and LC_90_ values recorded at 53.4% and 85.3%, respectively. The extract exhibited low residual activity and did not induce any phytotoxic effects on *J. curcas* plants (do Nascimento et al., 2018). A recent study conducted by Too et al. (2024) isolated trans-ferrulate and fatty-acid derivatives from *P. juliflora* leaves and identified them as main contributors to its insecticidal activity. Bioactive fractions showed strong efficacy against adult aphids adult aphids (*Brevicoryne brassicae*), producing 90–94% mortality in leaf-disc assays. The research done by Gousiga et al. (2025) showed that *P. juliflora* seedpod essential oil is rich in alkaloids, flavonoids, terpenoids, steroids, and phenolics and caused approximately 90% larval mortality. It also provided 76.6% protection against mosquitoes (*Aedes agepyti*) that highlights its potential as a natural alternative to chemical insecticides. According to Rodriguez Velazquez et al. (2025), the alkaloid-rich compounds in *P. juliflora* exhibit notable antiparasitic effects on equine gastrointestinal parasites in horses. Although direct studies on herbicidal properties in *S.* sp*ectabilis* and *A. mearnsii* remain limited, researchers recognize that their alkaloids and phenolic compounds could offer new opportunities. *S.* sp*ectabilis* exhibits strong allelopathic effects through its leaf extracts, significantly inhibiting the growth of plant species viz., *Vigna radiate*, *Cicer arietinum*, *Amaranthus cruentus*, *Bambusa bambos* (Prajitha and Bai, 2024). These findings suggest that IAPS could provide natural, eco-friendly solutions to reduce chemical pesticide use, while also helping to manage their spread across ecosystems.

## Fodder and nutritional aspects

Although invasive alien plant species (IAPS) often raise concerns due to their toxicity, some species provide valuable fodder resources under controlled use. *L. camara* plant contains high crude protein content, low fiber, and adequate macro-minerals for small ruminants, making it a potential browse species for goats in southern Africa (Ntalo et al., 2022). Worku and Tessema (2019) conducted an observational study to assess the nutritional composition of *L. camara* leaves and seeds as potential animal feed. However, it is known to be toxic to livestock due to pentacyclic triterpenoids, which can cause liver damage and photosensitivity. The leaf composition includes crude protein (23.3%), ash (15.7%), dry matter (88%), crude fat (4.4%), and total digestible nutrients (TDN) at 78.2%. Despite attempts to detoxify the leaves through sun-drying, boiling, and chemical treatment, cautious use is recommended and large-scale feeding should be avoided without proper processing. *P, juliflora* pods offer a rich carbohydrate (70.64%) (Ziara Hashim et al., 2025), 20 –30% sucrose and about 15% crude protein with a low concentrations of tannins and other unpalatable chemicals (Chharang, 2022). In several parts of Afar, Ethiopia, *P. Juliflora* has become a major source of dry season feed for goats, camels and donkeys (Fensham et al., 2024). de Melo Cavalcante et al. (2022) reported that *P. juliflora* grain flour is rich in protein and phenolics and showed the best functional and antioxidant properties. *L. leucocephala* stands out as a highly nutritious fodder option, containing 22–28% crude protein and showing a digestibility rate of up to 70%. However, the presence of mimosine limits its use in non-ruminants and young animals. Probiotic inoculation with *Synergistes jonesii* or ensiling techniques can break down mimosine and make *L. leucocephala* safer for feeding. These examples show that while IAPS offer important nutritional opportunities, careful management remains essential to minimize risks and maximize their value in livestock production.

## Prioritizing invasive alien plant species for value-added utilization

To systematically evaluate and prioritize invasive alien plant species (IAPS) for their diverse value-added applications, a comprehensive multi-criteria scoring approach was employed. Seven criteria were selected for evaluation: bioenergy potential, pulp and paper suitability, medicinal value, dye potential, toxicity (inverse), area of spread (inverse), and market readiness. Each criterion was chosen for its relevance to resource valorization and ecological or economic significance. Species were scored on a scale of 0 to 10 using a combination of literature review, expert judgment, and previously published data. Weights were assigned to each criterion to reflect their relative importance, with the highest weight given to bioenergy (0.20), followed by pulp (0.15), medicinal value (0.15), area (0.15), market readiness (0.15), dye (0.10), and toxicity (0.10). The weighted sum model (WSM) was applied to calculate a total score for each species using the formula:


$$Total\;Score=\sum_{i=1}^{n}\left(wi-si\right)$$

*wi*= weight of criterion *i*.

*si*= Score of the species under criterion *i*.

**Figure 4:**
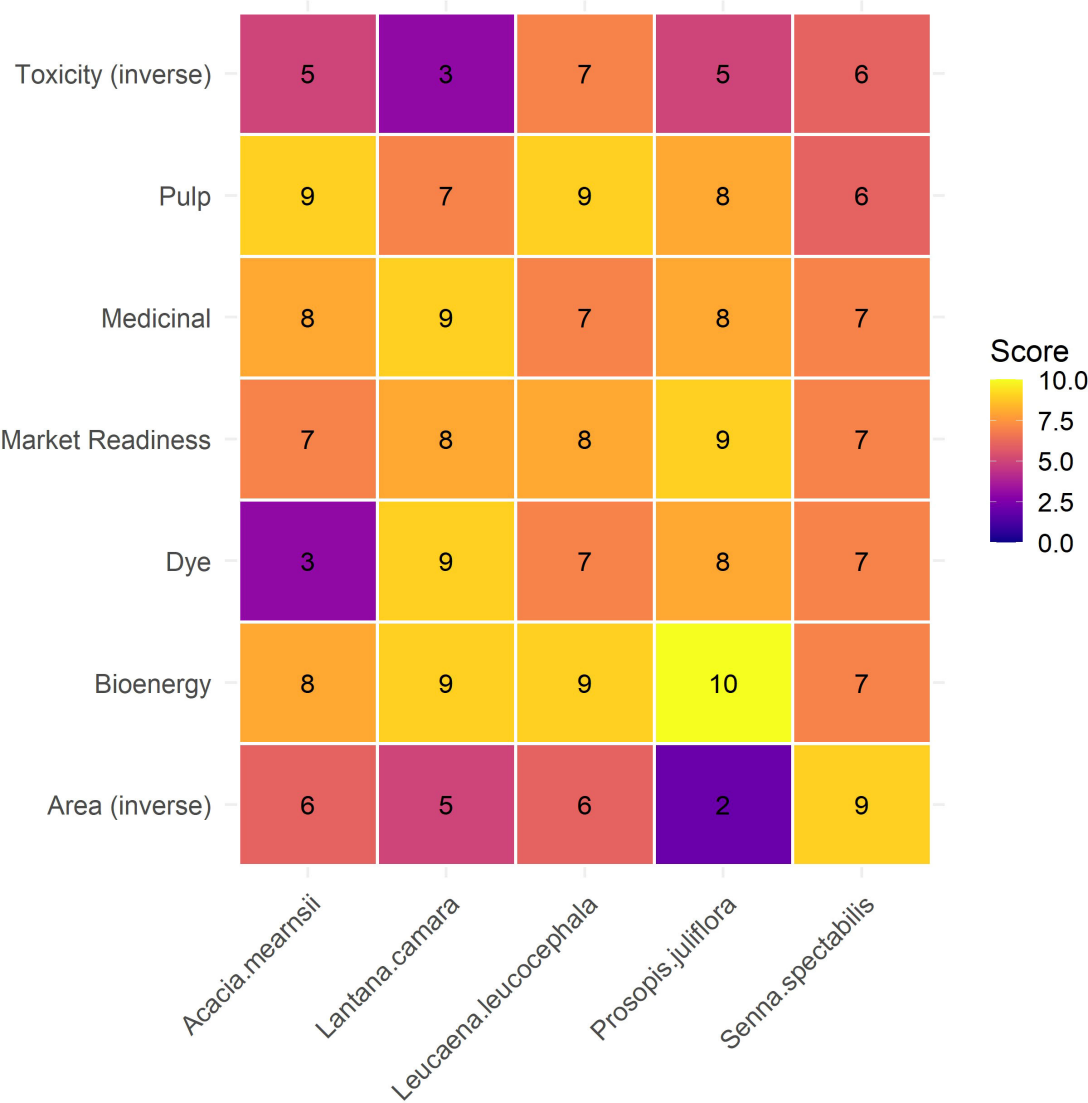
Multi-Criteria scoring of Invasive Alien Plant Species (IAPS) for utilization potential. This heatmap presents a comprehensive multi-criteria scoring of five invasive alien plant species (*A. mearnsii*, *L. camara*, *L. leucocephala*, *P. juliflora*, and *S.* sp*ectabilis*) across seven evaluation criteria: Toxicity (inverse), Pulp, Medicinal, Market Readiness, Dye, Bioenergy, and Area (inverse). The color gradient indicates the score, with darker green representing higher potential (or lower toxicity/area invaded). The chart highlights *P. juliflora* as a versatile species with consistently high scores across multiple categories, particularly in Bioenergy and Market Readiness, while *L. camara* and *L. leucocephala* show specialized potential in areas like natural dyes, medicinal uses, pulp, and fodder.

Higher total scores indicated greater overall suitability for circular utilization. The results were visualized using heatmap (**Figure 4**) to highlight comparative advantages among species and identify the most promising candidates for multipurpose valorization strategies. This robust methodology facilitates a quantitative assessment of each species’ potential across various valorization categories. Such an assessment is critical for identifying optimal pathways for resource utilization and for supporting the principles of a circular bioeconomy. This systematic prioritization is indispensable for guiding effective sustainable resource management, optimizing the allocation of efforts, and ultimately maximizing the multifaceted economic and ecological benefits derived from IAPS valorization initiatives.

## Challenges, opportunities and future perspectives

Using invasive alien plant species (IAPS) for value-added products brings both exciting opportunities and notable challenges. Scientists face significant obstacles, including inconsistent biomass availability, seasonal variability, and limited chemical composition data for many species. In some cases, the presence of toxic compounds, such as lantadenes, mimosine, and alkaloids, restricts their safe application in food, fodder, and pharmaceutical products. High lignin content and complex fiber structures also complicate their conversion into bioenergy and pulp unless advanced pretreatment methods are applied. Economic hurdles, including high processing costs and limited market access, slow down the commercial scaling of IAPS-derived products. Furthermore, unclear legal frameworks surrounding the harvesting and transport of invasive species create regulatory barriers.

Despite these difficulties, the potential benefits of valorizing IAPS remain compelling. Their fast growth, adaptability to degraded lands, and rich bioactive profiles open doors across industries such as bioenergy, natural dyes, pharmaceuticals, engineered wood products, and soil restoration. Research advancements in green chemistry, microbial biotechnology, and phytoremediation techniques continue to improve the feasibility of using IAPS biomass. Policy shifts that support invasive biomass utilization, coupled with incentives for circular economy practices, could create new markets and livelihood opportunities, especially in rural communities. Moving forward, researchers must focus on multidisciplinary innovation, process optimization, safe product development, and active community participation. Transforming IAPS from ecological threats into bioresource assets will require collaboration across science, industry, and policy at both local and global levels (**Supplementary Figures S1**–**S5**).

## Conclusion

This review redefines invasive alien plant species (IAPS) not merely as ecological threats but as valuable raw materials for circular bioeconomy applications. Through a combination of scoring analyses and literature synthesis, it was observed that *P. juliflora* stands out as the most multipurpose species, offering high potential in bioenergy, activated carbon, fodder, and even antimicrobial applications*. L. camara* excels in medicinal, dye, and furniture applications, but is limited by its toxicity to livestock. *L. leucocephala*, with its high digestibility and low lignin content, is well-suited for use as pulp, compost, and controlled fodder. *A. mearnsii* exhibits exceptional utility in pulping and water remediation, while *S.* sp*ectabilis* offers moderate potential across various categories, particularly in biochar production and allelopathic weed control.

Recommendations:

- Promoting *P. juliflora* and *L. leucocephala* in biorefineries as core IAPS feedstocks.
- Incentivizing local IAPS-based product development (e.g., dyes, charcoal, biopesticides).
- Integrating Multi-Criteria Decision Analysis (MCDA) tools, life-cycle thinking, and geospatial mapping in species selection.
- Establishing national guidelines for harvesting, processing, and marketing IAPS biomass.

The transformation of these plants from ecological threats into productive resources contributes to reducing their spread, strengthening rural economies, and minimizing dependence on forests. Future work should focus on techno-economic modeling, product certification, and participatory policy-making to fully unlock the bioeconomic potential of IAPS.
