# Supplementary figures and images for: Strategic valorization of invasive alien plants: A bioeconomic review for sustainable product development
Source: Front Plant Sci. 2026 Jan 13;16:1697102. doi: 10.3389/fpls.2025.1697102 (PMC12866983; doi:10.3389/fpls.2025.1697102)

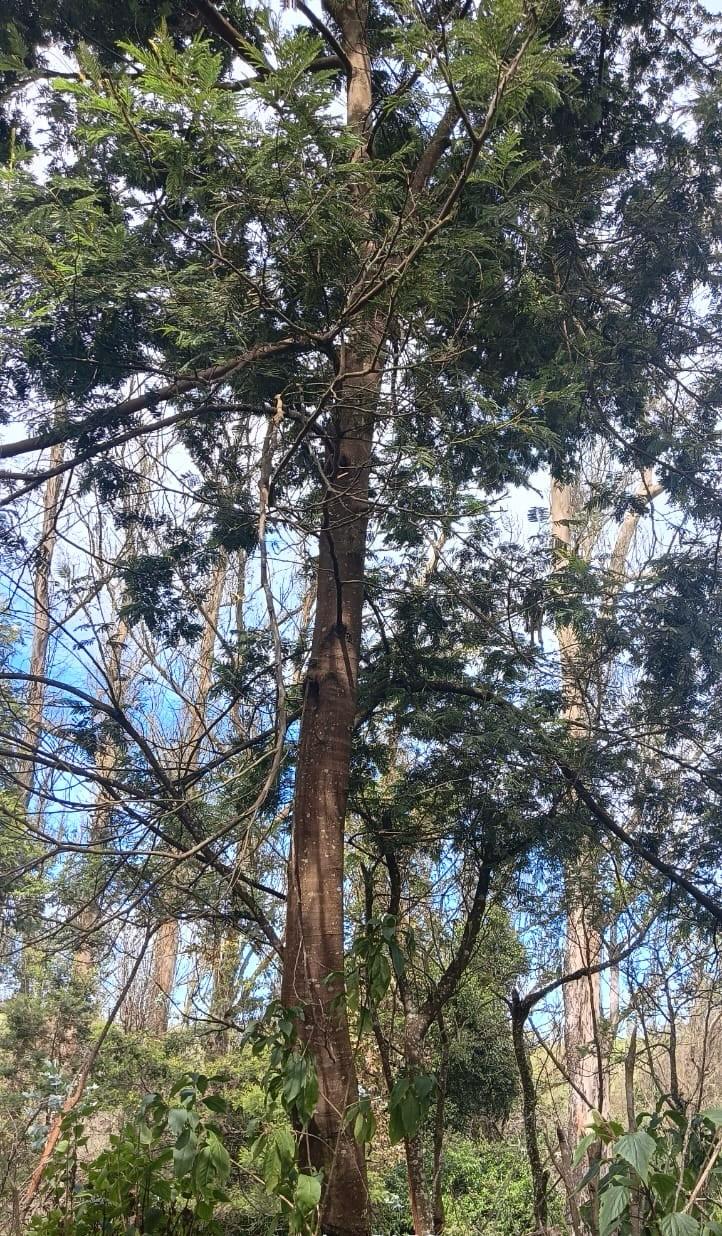

Supplement: Supplementary file 1 [file Image1.jpeg]

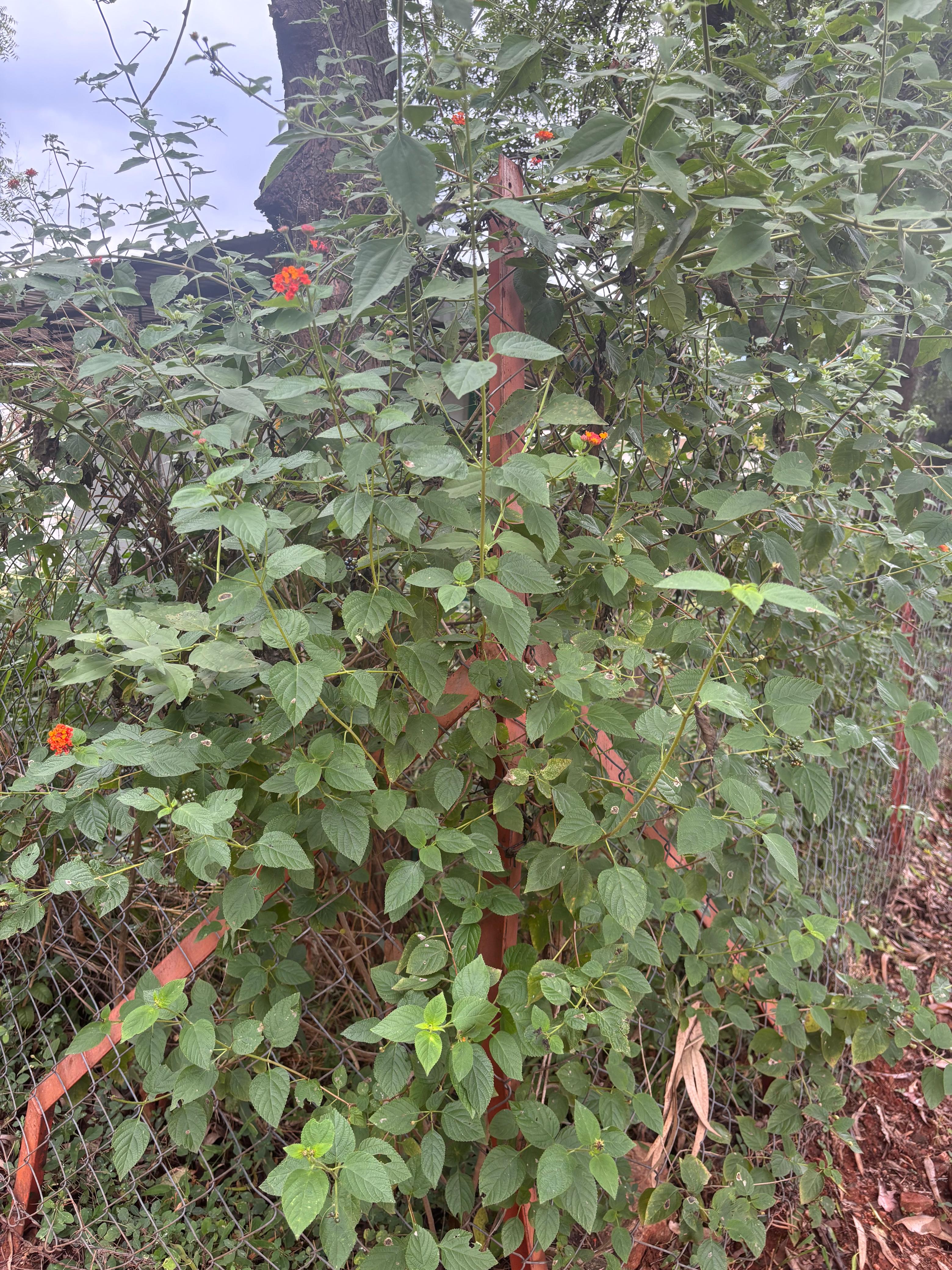

Supplement: Supplementary file 2 [file Image2.jpeg]

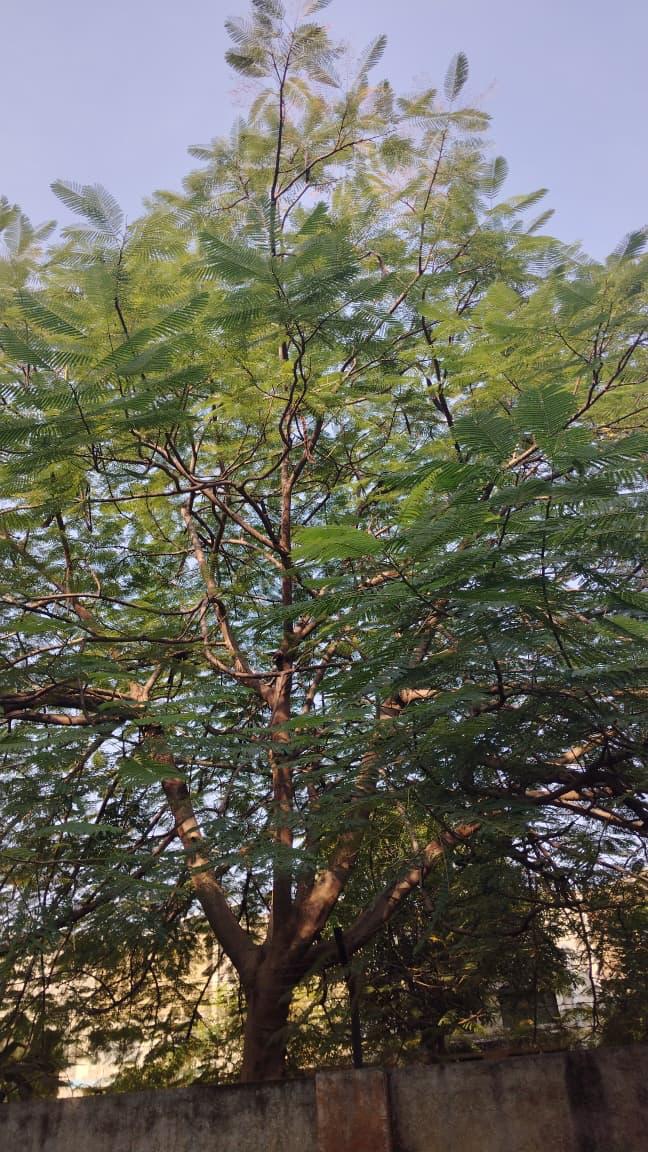

Supplement: Supplementary file 3 [file Image3.jpeg]

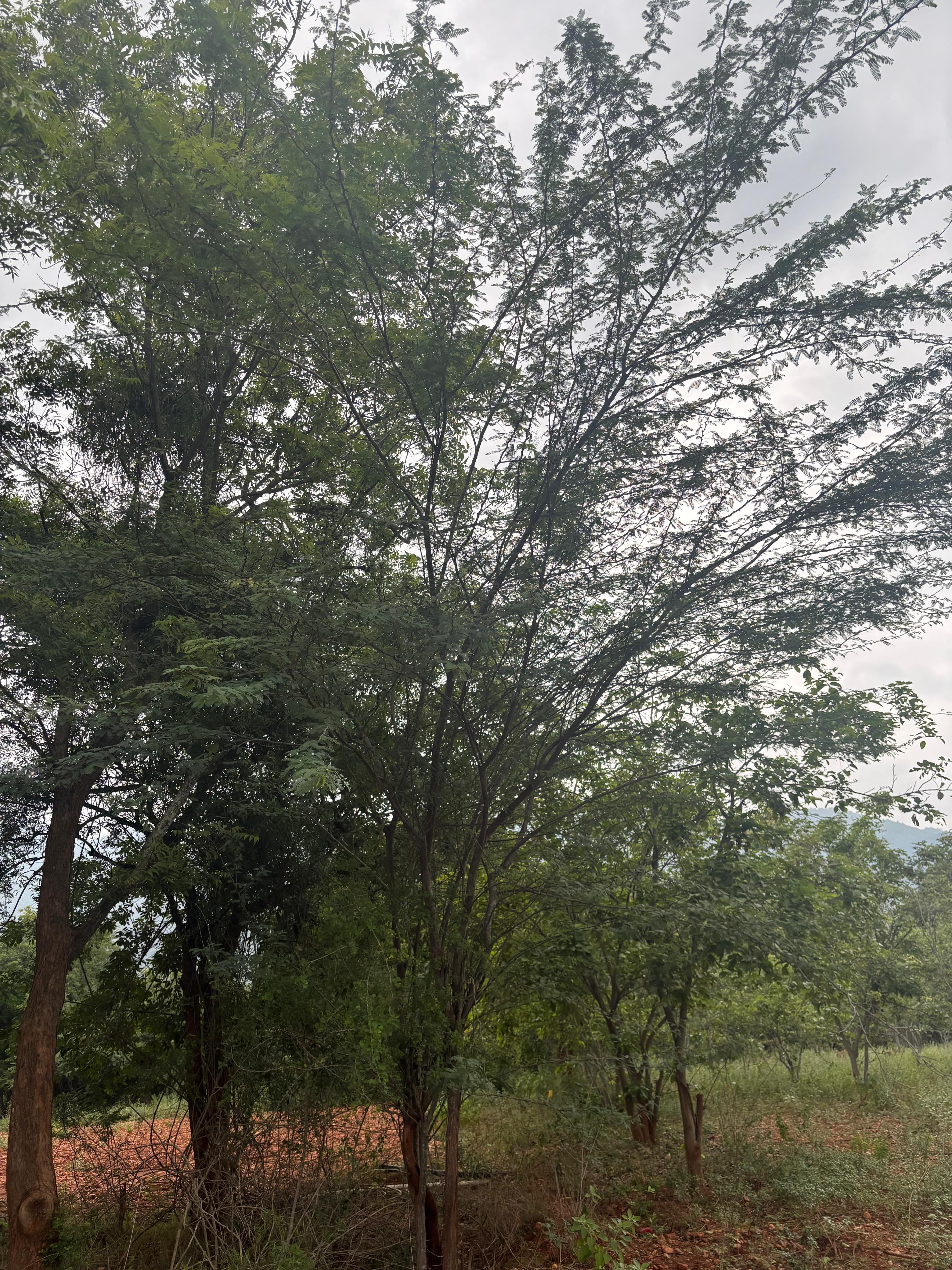

Supplement: Supplementary file 4 [file Image4.jpeg]

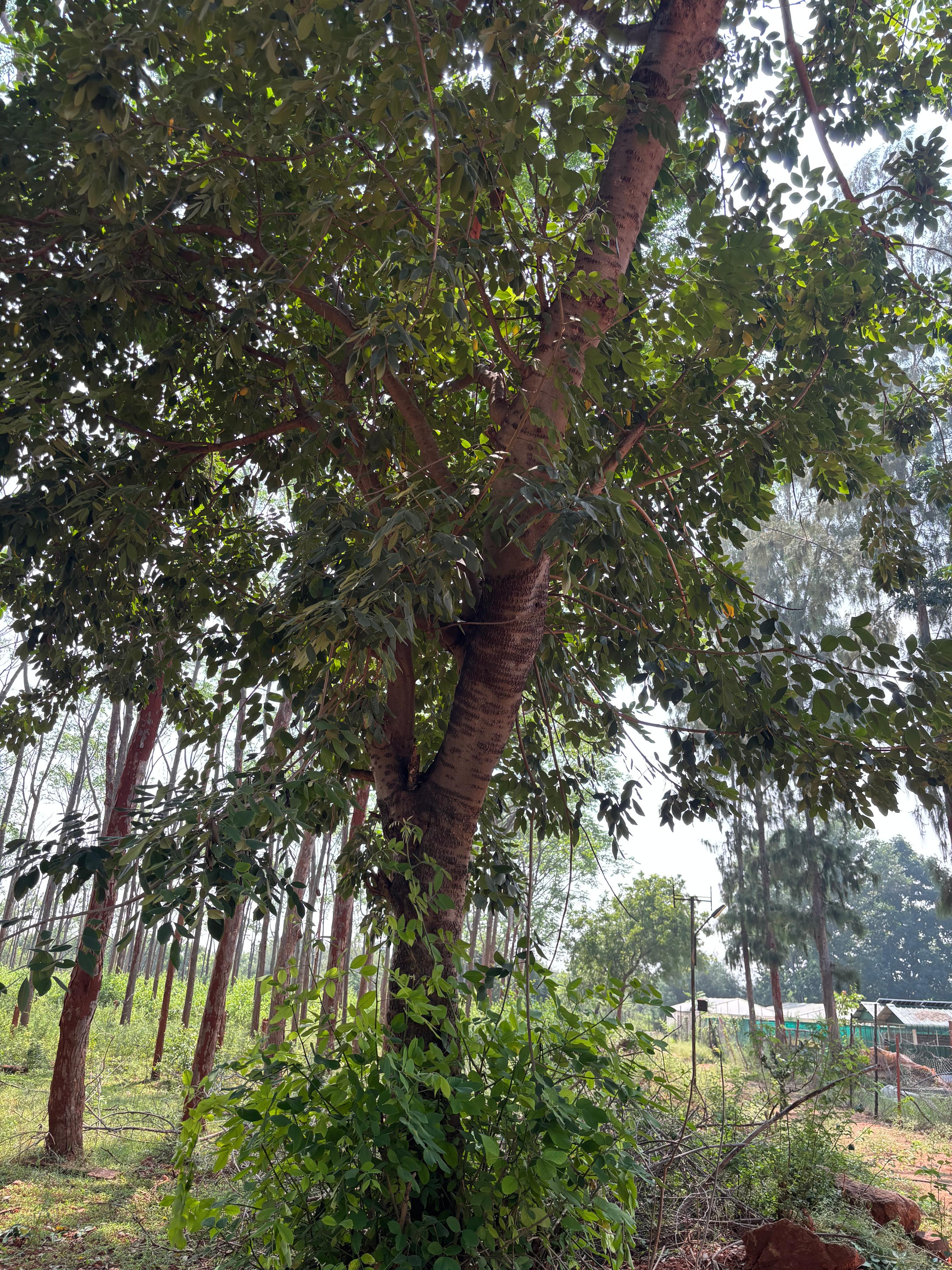

Supplement: Supplementary file 5 [file Image5.jpeg]
